# Haematopoietic ageing through the lens of single-cell technologies

**DOI:** 10.1242/dmm.047340

**Published:** 2021-01-22

**Authors:** Paulina M. Strzelecka, Frederik Damm

**Affiliations:** 1 Department of Hematology, Oncology, and Tumor Immunology, Charité - Universitätsmedizin Berlin, 13353 Berlin, Germany; 2 German Consortium for Translational Cancer Research (DKTK), 69120 Heidelberg, Germany; 3 Berlin Institute of Health, 10178 Berlin, Germany

**Keywords:** Haematopoietic ageing, Single-cell multiomics, Single-cell epigenomics, Single-cell transcriptomics

## Abstract

Human lifespan is now longer than ever and, as a result, modern society is getting older. Despite that, the detailed mechanisms behind the ageing process and its impact on various tissues and organs remain obscure. In general, changes in DNA, RNA and protein structure throughout life impair their function. Haematopoietic ageing refers to the age-related changes affecting a haematopoietic system. Aged blood cells display different functional aberrations depending on their cell type, which might lead to the development of haematologic disorders, including leukaemias, anaemia or declining immunity. In contrast to traditional bulk assays, which are not suitable to dissect cell-to-cell variation, single-cell-level analysis provides unprecedented insight into the dynamics of age-associated changes in blood. In this Review, we summarise recent studies that dissect haematopoietic ageing at the single-cell level. We discuss what cellular changes occur during haematopoietic ageing at the genomic, transcriptomic, epigenomic and metabolomic level, and provide an overview of the benefits of investigating those changes with single-cell precision. We conclude by considering the potential clinical applications of single-cell techniques in geriatric haematology, focusing on the impact on haematopoietic stem cell transplantation in the elderly and infection studies, including recent COVID-19 research.

## Abstract (precis)

**Summary:** We discuss the cellular changes that occur during haematopoietic ageing at the different molecular levels, and provide an overview of the benefits of investigating those changes with single-cell precision.

## Introduction

Since 2009, access to single-cell techniques has broadened from a few expert laboratories to research groups around the world (Lim et al., 2020). This shift was possible due to the commercialisation of these technologies (e.g. 10× Genomics, Pacific Biosciences, Illumina) and resulted in an increased application of single-cell techniques to study biological phenomena, from basic research (Canu et al., 2020; Farnsworth et al., 2020; Pijuan-Sala et al., 2020) through understanding intercellular interactions in the human body during homeostasis (James et al., 2020; Litviň uková et al., 2020; Park et al., 2020) to tracking disease-related changes (Martin et al., 2019; Nam et al., 2019; Vieira Braga et al., 2019). Haematology as a clinical discipline has been known as an early adopter of technological developments (Laurenti and Göttgens, 2018); therefore, it comes as little surprise that this branch of science quickly added single-cell techniques to its portfolio. Single-cell approaches provide the highest-resolution insight into the cellular makeup of complex systems possible, thus constituting a great tool for dissecting the dynamics of haematopoietic ageing. On a phenotypic level, haematopoietic ageing manifests through the loss of adaptive immunity, an increased rate of cytopaenias (see Glossary, Box 1) and an increased risk of developing myeloid malignancies (Gazit et al., 2008). Behind this phenotype lay molecular changes in the genome, transcriptome and epigenome. Single-cell technologies hold promise for a better understanding of ageing mechanisms by uncovering the changes happening in the body in a cell-dependent manner. This is important from a clinical pathology point of view, as connecting altered pathways with molecular changes in the context of cell types opens new avenues for possible pharmaceutical manipulation to reverse the ageing phenotype. In this Review, we focus on new insights gained from the application of single-cell technologies to study haematopoietic ageing over the past 5 years. The technical aspects of single-cell approaches are not covered here, as several excellent reviews on this topic are already available (Clark et al., 2016; Gawad et al., 2016; Mincarelli et al., 2018; Svensson et al., 2018). However, for a short overview of each technique please refer to Box 2.

**Box 1. Glossary**

**Clonal repertoire of adaptive lymphocytes:** adaptive lymphocytes (T and B cells) are characterised by the presence of antigen-specific receptors, T cell receptors (TCRs) and B cell receptors (BCRs), respectively. Binding of an antigen to the receptor triggers an immune response. Cells from the same clone share the sequence of the receptor. Different clones have different antigen specificity. Therefore, higher variability of the clonal repertoire (T and B cells with distinct TCR/BCR sequences) provides protection against more pathogens.

**CpG islands:** parts of the genome characterised by the presence of large numbers of CG dinucleotide repeats.

**Cytopaenia:** deficiency in the number of mature blood cells.

**Engraftment:** ability of transplanted haematopoietic stem cells (HSCs) to contribute to the stable blood production in a new host upon transplantation.

**Homing:** ability of HSCs to migrate to bone marrow (BM), where they can contribute to the production of new blood cells.

**Immunosenescence:** changes in the immune system caused by ageing and resulting in the deterioration of the immune response.

**Inflammaging:** chronic low-grade inflammation typical of ageing.

**Lineage bias:** increased production of one or more blood lineages at the expense of the other lineages (e.g. aged HSCs were shown to produce more myeloid cells and fewer lymphoid cells compared to their young counterparts).

**Long-term HSCs (LT-HSCs):** subset of HSCs responsible for the long-term production of blood cells.

**Peripheral blood mononuclear cells (PBMCs):** population of blood cells with single nuclei (monocytes, lymphocytes, dendritic cells).

**Poly(dT) primer:** single-stranded sequence of deoxythymidine (dT), which anneals to the poly(A) tail of mRNA molecules and is used during the reverse transcription reaction.

**Pseudotime analysis:** method of single-cell trajectory analysis, which measures the progression of a cell through a biological process, e.g. differentiation.

**Quiescence:** ability of HSCs to stay dormant (not to divide).

**Self-renewal:** ability of HSCs to give rise to new HSCs without differentiation.

**Box 2. Overview of single-cell techniques**

**Single-cell DNA sequencing (scDNAseq):** provides information about the DNA content of single cells. The most common parameters investigated using this approach include single-nucleotide variants (Lodato et al., 2015) and copy number variations (Knouse et al., 2016).

**Single-cell RNA sequencing (scRNAseq):** provides information about the transcriptome profile of a single cell. Polyadenylated mRNA is separated using oligo-dT primers and converted into complementary DNA (cDNA), followed by amplification, library construction and sequencing. Currently available scRNAseq methods can be roughly divided into plate-based and droplet-based techniques. One of the main differences between those techniques is throughput: the plate-based approach allows the analysis of up to hundreds of cells, whereas droplet-based assays interrogate up to tens of thousands of cells (Haque et al., 2017; Knouse et al., 2016).

**Single-cell transposase-accessible chromatin sequencing (scATACseq):** a method to study genome-wide chromatin accessibility in single cells. It allows the identification of regulatory regions of open chromatin throughout the genome. The method uses Tn5 transposase and inserts sequencing primers into regions of open chromatin (Chen et al., 2018).

**Single-cell chromatin immunoprecipitation sequencing (scChIPseq):** analyses DNA-protein interactions. Combination of chromatin immunoprecipitation with DNA sequencing allows the identification of binding sites of DNA-associated proteins. This method can capture repressive and active chromatin states (Grosselin et al., 2019; Rotem et al., 2015).

**Single-cell bisulphite sequencing (scBSseq):** provides information about the methylation profile of single cells. It measures DNA methylation at cytosine residues (5mC), an epigenetic mark critical in the regulation and maintenance of cell-type-specific transcriptional programs (Smallwood et al., 2014).

**Single-cell mass cytometry (CyTOF):** this method allows the analysis of the protein content of single cells using a combination of flow cytometry and mass spectrometry. CyTOF uses antibodies conjugated to rare heavy metal isotopes and measures its time of flight (TOF), a parameter characteristic to the atom's mass. The use of metal isotope-conjugated antibodies in lieu of fluorophore-conjugated ones overcomes the problem of spectral overlap in multicolour flow cytometry and allows for the measurements of over 40 parameters simultaneously (Gadalla et al., 2019; Kay et al., 2016).

**Single-cell multiomics:** the combination of usually two single-cell techniques (for example, scRNAseq and scATACseq) (Granja et al., 2019) to investigate multiple molecular features in the same cell (Hu et al., 2018).

## Haematopoietic ageing

**Fig. 1.**
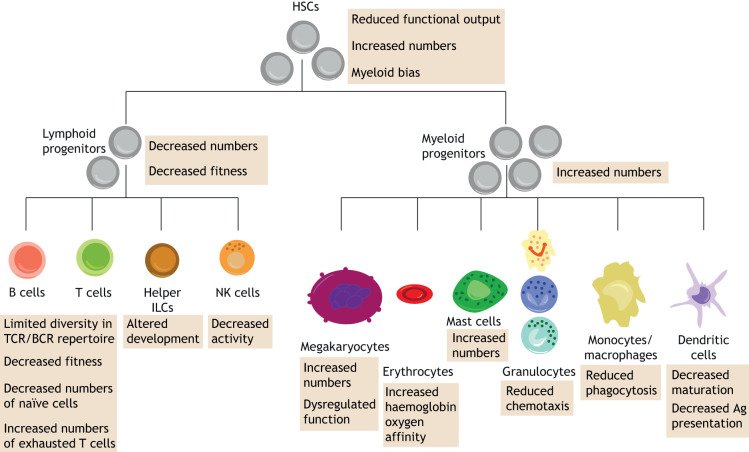
**Age-related changes affecting haematopoietic hierarchy.** Age-related changes have different effects on haematopoietic cells depending on their lineage identity. Aged HSCs are characterised by decreased functional output, which is compensated for by their increased numbers. They also display myeloid bias (generating reduced numbers of lymphoid progeny). Haematopoietic ageing affects mature blood cells in a cell-type-specific manner. Depending on the cell type, it can impact cell numbers (B and T lymphocytes, megakaryocytes, mast cells), functional activity (B and T lymphocytes, NK cells, megakaryocytes, erythrocytes, granulocytes, monocytes/macrophages, dendritic cells) or development (helper ILCs, dendritic cells). Ag, antigen; BCR, B cell receptor; HSC, haematopoietic stem cell; ILC, innate lymphoid cell; NK, natural killer; TCR, T cell receptor.

Ageing leads to numerous changes that affect multiple components of the body. The haematopoietic system consists of blood cells and their niche, the bone marrow (BM). The alterations in BM and haematopoietic stem cells (HSCs) caused by ageing disturb tissue homeostasis. A key discovery in HSC ageing research revealed that HSCs from old mice had only 25% of the efficiency of young HSCs in terms of homing (Box 1) and engraftment (Box 1) into the BM of a transplant recipient (Morrison et al., 1996). Since then, it has been shown that aged HSCs have reduced functional output, which is compensated for by their increased numbers (de Haan et al., 1997). Aged HSCs also have reduced lymphoid potential, whereas their myeloid potential (particularly granulopoiesis) is increased (Rossi et al., 2005; Sudo et al., 2000) (Fig. 1). Similarly, age-related changes also affect the BM. Some factors from the BM microenvironment (such as cytokines and enzymes) are crucial contributors to the ageing process (Frisch et al., 2019). Aged BM has a reduced capability of retaining HSCs in the niche, and increased mobilisation of HSCs was observed in older mice (Xing et al., 2006). Thus, changes related to ageing can be roughly classified as intrinsic (occurring within the blood cells themselves) and extrinsic (affecting the BM microenvironment). Single-cell technologies are increasingly contributing to a better understanding of the molecular mechanisms underlying haematopoietic ageing. Early studies within the field focused on an HSC-centric view of ageing (Grover et al., 2016; Kowalczyk et al., 2015) and are currently shifting towards a new emerging concept, which looks at haematopoietic ageing from the microenvironmental angle (Dorshkind et al., 2019; Ho et al., 2019; Maryanovich et al., 2018; Pioli et al., 2019). In general, haematopoietic studies applying single-cell technologies in the context of haematopoietic ageing can roughly be divided into four main areas: (1) lineage biases (Box 1) (Grover et al., 2016), (2) clonal expansion (Rodriguez-Fraticelli et al., 2020), (3) epigenetic signature (Cheung et al., 2018; Florian et al., 2018) and (4) niche-related changes (Baccin et al., 2020; Oetjen et al., 2018; Silberstein et al., 2016; Tikhonova et al., 2019) (Table 1).

**Table 1.**
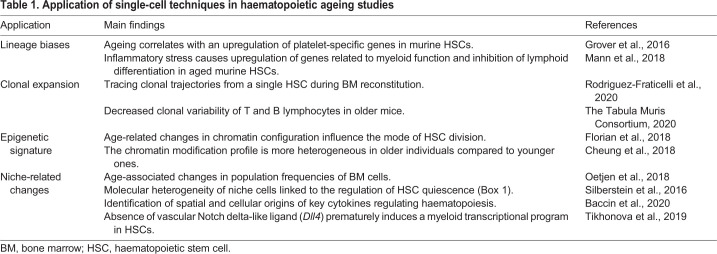
**Application of single-cell techniques in haematopoietic ageing studies**

## Single-cell techniques in geriatric haematology

Geriatric haematology is a great example of a clinical research field that can benefit from dissecting the changes that occur during haematopoietic ageing with single-cell precision. In this context, advances in single-cell techniques can shed new light on the dynamics of blood cell reconstitution upon transplantation in elderly recipients (Frick et al., 2019; Rodriguez-Fraticelli et al., 2020), provide information on how to improve immune system function and decrease the risk of developing age-associated myeloid malignancies (Giladi and Amit, 2018; Paguirigan et al., 2015), identify biomarkers of ageing (Ma et al., 2020) or help to develop rejuvenation strategies (Li et al., 2020).

## Age-related changes in DNA

Accumulation of DNA damage is considered a hallmark of ageing. However, establishing the causal relationship between ageing and somatic mutations remains challenging. This is a field in which single-cell DNA sequencing can be successfully applied.

In general, most somatic mutations are characterised by their random nature and low abundance; therefore, population-level studies would predominantly highlight the germline genotype instead of *de novo* somatic mutations. Numerous somatic mutations accumulate during ageing. As blood is one of the most proliferative systems in the human body (Gondek and DeZern, 2020), it is also prone to acquiring somatic mutations at a higher rate. The presence of certain somatic mutations in HSCs adds to their fitness advantage and results in the clonal expansion of mutation-positive HSCs, which contributes to clonal haematopoiesis (Jaiswal et al., 2014). In healthy young individuals, all HSCs are equally capable of producing all of the mature blood cells, which maintains polyclonal haematopoiesis (Doulatov et al., 2012). By contrast, during age-related clonal haematopoiesis, a substantial proportion of mature blood cells is derived from a dominant HSC clone. It is estimated that at least 30% of all elderly people are affected by clonal haematopoiesis (Jaiswal et al., 2014; McKerrell et al., 2015; Young et al., 2016). The presence of clonal haematopoiesis is linked to an elevated risk of haematological malignancies and cardiovascular disease, contributing to an increased mortality rate overall (Steensma, 2018). Therefore, an in-depth understanding of somatic mutations that lead to clonal haematopoiesis is crucial (Arends et al., 2018; Mylonas et al., 2020; Ortmann et al., 2015), especially since it has been shown that the order in which mutations are acquired can influence the phenotypic manifestation of the pathology, including the response to targeted therapy and the biology of stem and progenitor cells (Ortmann et al., 2015).

### Somatic mutations at single-cell resolution

Several methods that allow investigation of somatic mutations at single-cell level are now available (Albertí-Servera et al., 2020; Lareau et al., 2020; Nam et al., 2019; Rodriguez-Meira et al., 2019; Zhang et al., 2019). TARGET-seq combines single-cell whole transcriptome analysis with single-cell targeted locus genotyping, thus enabling researchers to link mutation status with a gene expression profile in heterogeneous tumour cell populations (Rodriguez-Meira et al., 2019). Similarly, application of single-cell DNA sequencing helped to reveal clonal heterogeneity of acute leukaemias (Albertí-Servera et al., 2020; Miles et al., 2020; Morita et al., 2020). Finally, single-cell methods that use mutations within mitochondrial DNA to infer clonal relationships between cells have also been reported (Lareau et al., 2020; Ludwig et al., 2019). Single-cell studies investigating somatic mutations in blood cells focus on the characterisation of mutational signatures and their impact on cellular functions (Nam et al., 2019; Zhang et al., 2019). Application of single-cell whole-genome sequencing has already provided detailed information about somatic mutations that affect B cells during the human lifespan (Zhang et al., 2019). This approach has allowed researchers to distinguish mutational signatures specific to development from age-related ones. It revealed that an age-related mutational signature correlates with the signature of B cell leukaemia, highlighting the role of ageing as a cancer risk factor (Zhang et al., 2019). Single-cell techniques can be also applied to investigate the functional consequences of somatic mutations. This is possible when the presence of a mutation affects a protein-coding region of the mRNA sequence (Nam et al., 2019; van Galen et al., 2019). The Landau group developed a novel Genotyping of Transcriptomes (GoT) method, which allows the distinguishing of mutation-positive from mutation-negative cells within the same cell type (Nam et al., 2019). Application of this method enables the direct comparison of transcriptional profiles between cells of the same identity (e.g. monocytes). It thus gives a unique opportunity to pinpoint the alterations in gene expression stemming from the presence of the mutation. Recent studies implicate an altered inflammation profile of monocyte-derived macrophages as one of the factors contributing to cardiovascular disease in elderly people with clonal haematopoiesis (Jaiswal and Ebert, 2019). Application of GoT in clonal haematopoiesis studies will provide an in-depth understanding of how the presence of such mutations affects inflammatory pathways. This knowledge could later be translated into new pharmaceutical strategies to alleviate the risk of cardiovascular disease in individuals affected by clonal haematopoiesis.

## The transcriptome during ageing

Transcriptome analysis of different cell types provides valuable insight into their functional properties linked to their particular role in the body. Additionally, changes in a cell's microenvironment (such as inflammation) can also be detected as specific changes within the transcriptome (e.g. increased production of anti-inflammatory cytokines) (Butler et al., 2018; Hernández et al., 2018). Thus, the readout of the transcriptome is a good reflection of the cell state. Comparison of the transcriptomes between ‘young’ and ‘old’ cells within the same cell type coupled with functional assays therefore constitutes an excellent tool to dissect age-related changes in a cell's behaviour.

### Age-related gene expression changes in HSCs

Among single-cell studies, large-scale single-cell transcriptomic projects are leading the way in providing valuable insight into haematopoietic ageing (Grover et al., 2016; Kowalczyk et al., 2015; Mann et al., 2018). One of the advantages offered by single-cell-level analysis in those settings is the power to determine whether alterations occurring during ageing are homogeneously distributed across each cell population or rather specific to a subset of cells within the population. Even before the single-cell era, the decline of HSC function during ageing was well documented (de Haan et al., 1997; Rossi et al., 2005; Sudo et al., 2000); however, the underlying molecular mechanisms were not yet fully understood. Single-cell RNA sequencing (scRNAseq) deciphers the gene expression changes in individual HSCs associated with the ageing phenotype, such as lineage bias, imbalance between self-renewal (Box 1) and differentiation, and declined engraftment in transplantation assays (Dykstra et al., 2011). The landmark studies in the field are the studies of Kowalczyk et al. (2015) and Grover et al. (2016), which were among the first ones investigating the differences between young (2- to 3-month-old) and old (>22-month-old) murine HSCs at single-cell resolution. These studies provided information about the transcriptional mechanisms behind the imbalance in blood cell production observed in ageing. This insight into gene expression showed that young and old HSCs are transcriptionally different enough to form separate clusters during the analysis. The most prominent differences were observed in the expression of cell cycle-related genes and genes characteristic for the megakaryocyte/platelet lineage (Grover et al., 2016; Kowalczyk et al., 2015). Interestingly, old long-term HSCs (LT-HSCs) (Box 1) were significantly under-represented among cells in the G1/S cell cycle phase, suggesting a more rapid transition through this phase (Kowalczyk et al., 2015). It has been established that self-renewing cells have a shorter G1 phase (Coronado et al., 2013; Li et al., 2012). Therefore, this observation provides a possible mechanistic explanation behind the increased numbers of HSCs in aged BM (de Haan et al., 1997). ScRNAseq has also explained changes underlying the observed myeloid bias and decreased lymphoid potential of old HSCs (Fig. 1) (Rossi et al., 2005; Sudo et al., 2000). Comparison of individual HSC transcriptomes isolated from old and young mice showed that ageing correlated with an upregulation of platelet-specific gene expression to the point that the majority of aged HSCs almost exclusively produced platelets (Grover et al., 2016). The lymphoid potential of aged HSCs was successfully restored by knocking out the zinc finger protein, multitype 1 (*Zfpm1*) gene, which is involved in erythroid and megakaryocytic cell differentiation. Those findings illustrate the power of single-cell transcriptome analysis in highlighting possible intervention strategies for reversing an aged phenotype.

### Age-dependent inflammatory response of HSCs

Apart from investigating age-related changes in HSCs during homeostasis, scRNAseq has also provided insight into the age-dependent inflammatory response of HSCs (Mann et al., 2018). Old LT-HSCs display unique biases upon immunological stimulation. Under inflammatory stress, HSCs isolated from elderly mice switch to a myeloid-biased expression programme (upregulation of genes related to myeloid function and inhibition of lymphoid differentiation). By contrast, stimulated young HSCs upregulate genes related to lymphocyte development (adaptive immune response) and acute inflammatory response. Aged mice demonstrate a strong acute increase in myeloid output (innate immune response) following the inflammatory challenge, which was not observed in young mice (Mann et al., 2018). Those results are in line with the decreased competency of the adaptive immune system observed in the elderly (Fuentes et al., 2017), which relies mainly on B and T lymphocytes.

### Age-related gene expression changes in immune cells

Age-related changes in the transcriptome of single mature blood cells can also explain their altered functional properties observed at later stages of life. This is especially valuable in the study of the ageing immune system. Elderly people have a declining immune system, which manifests as an increased vulnerability to infectious diseases, diminished responses to vaccination and susceptibility to age-related inflammatory diseases (Aiello et al., 2019). Whole-blood and peripheral blood mononuclear cells (PBMCs) (Box 1), collected from healthy donors in a wide range of age groups, have been used to investigate the age-associated decline in immunity (Ademokun et al., 2010; Dunn-Walters and Ademokun, 2010; Sansoni et al., 1993). In line with that goal, scRNAseq is helping to better understand the mechanisms of immunosenescence and inflammaging (Box 1) in blood cells. A recent study from the Tabula Muris Consortium applied scRNAseq to create a comprehensive atlas of age-associated changes in different murine organs and tissues (The Tabula Muris Consortium, 2020). Their findings revealed that old leukocytes have increased expression of pro-inflammatory markers (*Cd14*, *Lgals3*, *Tnfrsf12a*) and decreased expression of anti-inflammatory markers (*Cd9*, *Cd81*), an observation consistent with the chronic low-grade inflammation typical of ageing. Additionally, analysis of the clonal repertoire of adaptive (T and B) lymphocytes (Box 1) from single-cell data pointed at a decreased clonal variability in older mice (Fig. 1) (The Tabula Muris Consortium, 2020). This explains, in part, the higher vulnerability of the elderly to infections [e.g. influenza, severe acute respiratory syndrome coronavirus 2 (SARS-CoV-2)] (Mueller et al., 2020) and lower benefits from vaccination reported in older individuals (Goronzy and Weyand, 2013). Another interesting study applied scRNAseq to investigate the population of peripheral blood cells from supercentenarians (people who reached at least 110 years of age) to understand the pathways and mechanisms that promote healthy ageing (Hashimoto et al., 2019). Supercentenarians display a delayed onset of age-related diseases (Andersen et al., 2012); therefore, they are considered a model of successful ageing. The analysis revealed the existence of a specific hybrid population of T cells, combining the features of helper and cytotoxic T cells. This discovery would not be possible in experiments on a population level. The existence of such hybrid T cells might be considered a specific adaptation that results in exceptional longevity (Hashimoto et al., 2019). Further single-cell studies focusing on generating an in-depth understanding of the mechanisms sustaining the development of these cells can benefit the modern ageing society.

## The epigenome during ageing

**Fig. 2.**
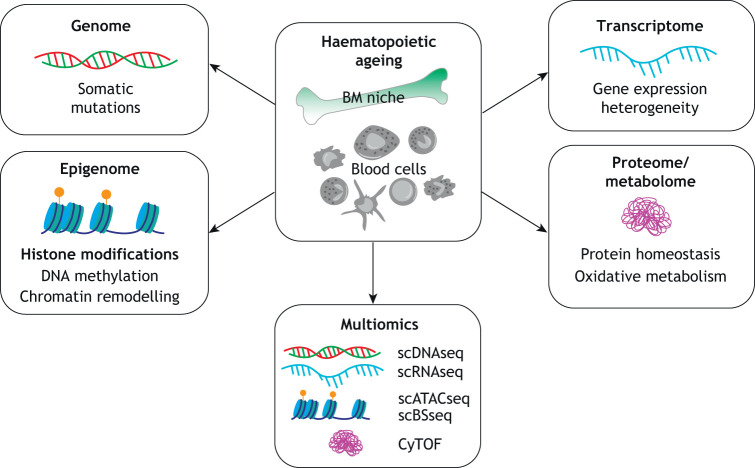
**Molecular changes related to haematopoietic ageing at different molecular levels.** Single-cell technologies provide insight into cell-to-cell variation in the context of haematopoietic ageing at various molecular levels. Current single-cell techniques enable the investigation of somatic mutations, epigenetic changes, gene expression heterogeneity and changes in the proteome and metabolome related to the process of ageing. The combination of different single-cell techniques (multiomics) allows for the investigation of multiple molecular features from the same cell, thus providing more data to understand the process of haematopoietic ageing. Yellow circles indicate methylation. BM, bone marrow; CyTOF, single-cell mass cytometry; scATACseq, single-cell assay for transposase accessible chromatin sequencing; scBSseq, single-cell bisulphite sequencing; scDNAseq, single-cell DNA sequencing; scRNAseq, single-cell RNA sequencing.

The ageing process is also responsible for the alteration of the chromatin structure and the epigenetic signature within HSCs. Epigenetics refers to all mechanisms regulating gene expression independent of the DNA sequence (Pagiatakis et al., 2019). Epigenetic changes associated with ageing include changes in DNA methylation, reorganisation of chromatin (Moskowitz et al., 2017; Ucar et al., 2017) and post-translational modifications of histones (Pal and Tyler, 2016) (Fig. 2). Over the past decade, several studies strongly implicated epigenetic mechanisms in the dysregulation of gene expression that is observed during the ageing process (Pagiatakis et al., 2019). Ageing in somatic tissues is linked to global hypomethylation of DNA (Gonzalo, 2010). Regulators of DNA methylation include DNA methyltransferases, which drive methylation of CpG islands (Box 1), and the ten-eleven translocation (Tet) enzymes, which regulate demethylation (Pan et al., 2017; Rodríguez-Ubreva et al., 2017). Expression of both types of enzymes differs between young and old HSCs (Beerman et al., 2013; Sun et al., 2014). Post-translational histone modifications can also disturb gene expression patterns in aged HSCs. Aged HSCs display broad peaks of H3K4me3 signal around self-renewal genes (Beerman et al., 2013; Sun et al., 2014), which are associated with higher gene expression (Dhar et al., 2018). Studying the epigenome plays a crucial role in age-related research, because it has been shown that DNA methylation can be used as a reliable biomarker of biological age, thus constituting a so-called ‘epigenetic clock’ (Horvath and Raj, 2018). For epigenomic profiling at the single-cell level, researchers can choose from single-cell assay for transposase accessible chromatin sequencing (scATACseq) (Chen et al., 2018; Satpathy et al., 2019), single-cell chromatin immunoprecipitation sequencing (scChIPseq) (Grosselin et al., 2019), single-cell bisulphite sequencing (scBSseq) (Smallwood et al., 2014) or single-cell reduced-representation bisulphite sequencing (scRRBS) (Guo et al., 2013). Examining epigenome-related changes in the context of haematopoietic ageing is appealing, as an investigation of potential biological ageing estimators showed the superiority of epigenetic clock estimations (Jylhävä et al., 2017). As epigenetic ageing manifests via alterations that affect a minute number of cells, it is perfect for the application of single-cell techniques (Horvath and Raj, 2018).

### Influence of chromatin structure on HSC division

With recent developments in scATACseq scalability allowing it to be used in both droplet-based mode (interrogates up to tens of thousands of cells) (Satpathy et al., 2019) and plate-based mode (allows the analysis of up to hundreds of cells) (Chen et al., 2018), its use to decipher specific cellular mechanisms increases (Popescu et al., 2019; Ranzoni et al., 2020). Recently, scATACseq analysis revealed the involvement of chromatin structure in executing different modes of HSC division (Florian et al., 2018). Old HSCs were shown to divide symmetrically, supporting self-renewal. By contrast, young HSCs preferentially underwent asymmetric division, generating progenitor cells (Florian et al., 2018). This yet again provided a direct link to the increased number of HSCs in the BM upon ageing (de Haan et al., 1997). Solely based on transcriptome profiles obtained from scRNAseq experiments, it was not possible to predict the mode of the division. Only insight into the open chromatin configuration obtained from the analysis of scATACseq peaks showed the correlation between the mode of division and HSC age, and allowed the prediction of the fate of the daughter cell (retaining stem cell potential or differentiating) (Florian et al., 2018). This supports the importance of multimodal measurements on single cells to decipher the mechanisms underlying the altered phenotype in ageing.

### Chromatin profile of aged immune cells

Another study employed a single-cell method based on mass cytometry, epigenetic landscape profiling using cytometry by time-of-flight [EpiTOF; a variation of single-cell mass cytometry (CyTOF; Box 2)], to look at the chromatin modification profiles across immune cells during ageing (Cheung et al., 2018). This method allows the measurement of diverse chromatin marks in single cells and reveals an increased cell-to-cell variability in chromatin marks as a signature of immune cell ageing. Chromatin modification profiles in younger individuals (<25 years old) are homogeneous, whereas those of older individuals (>65 years old) are heterogeneous. Interestingly, an analysis of cells from monozygotic twins pointed out that these variations are largely caused by non-heritable factors (Cheung et al., 2018). This single-cell approach contributed to the creation of a comprehensive immune cell epigenetic atlas of age-associated changes based on chromatin modification profiles. Thus, the above examples illustrate how application of single-cell epigenomics provides a deeper understanding of the mechanisms behind the regulation and maintenance of the aberrant gene expression that is observed in ageing.

## Ageing and metabolomics research

Metabolomics is one of the novel approaches in systems biology with a high potential to provide important information about the ageing process. Metabolites, the end products of a biological process, provide a quick readout of the function-related phenotype. The metabolic composition of the cell is strictly dependent on physiological conditions (Onjiko et al., 2015). Studies in animal models show that calorie restriction, which directly influences metabolism, is an effective method against age-related diseases (Balasubramanian et al., 2017). Additionally, glucose metabolism has been shown to influence chromatin structure and the transcription process (Heiden et al., 2009), and to interfere with stem cell fate decisions in terms of proliferation, differentiation and dormancy (Shyh-Chang et al., 2013). Therefore, applying metabolomics to study haematopoietic ageing can provide an additional layer of understanding of this process.

### The influence of fasting on the ageing process

Recently, a new study provided an atlas of single-cell transcriptomic changes that are associated with the influence of fasting on the ageing process. It demonstrated how metabolic intervention can rewire ageing programmes in multiple cell types, including blood cells and the BM (Ma et al., 2020). Calorie restriction successfully replenishes the number of certain blood cells in the BM (including precursors of B cells and erythrocytes, T cells and dendritic cells) that are reduced during ageing (Ma et al., 2020). Additional pseudotime analysis (Box 1) also revealed that, during ageing, neutrophils acquire a migratory phenotype and infiltrate peripheral tissue, thus explaining the inflammaging phenotype. This migration was reversed by a calorie restriction strategy (Ma et al., 2020).

### Glycolysis in aged haematopoietic progenitor cells (HPCs)

The combination of scRNAseq with metabolomic and proteomic approaches has provided interesting insights into the behaviour of aged HPCs (Hennrich et al., 2018; Poisa-Beiro et al., 2020). Aged HPCs with myeloid bias potential displayed elevated glycolysis (Hennrich et al., 2018). The increase in glycolytic enzyme levels was attributed to the expansion of HPC subpopulations that become more glycolytic upon ageing (Poisa-Beiro et al., 2020). Even though these findings derive from the metabolomics analysis at the population level, single-cell metabolomics holds promise to further advance haematopoietic ageing research. Single-cell metabolomics is still under development, but already constitutes an exciting research area. This approach is particularly challenging, as mass spectrometry methods struggle with the identification of short-lived biomolecules that are not chemically stable *in vitro*. Nevertheless, there are reports of the successful detection of small amounts of metabolites from single cells (Comi et al., 2017; Duncan et al., 2019; Zhang and Vertes, 2018). In the future, it is likely that the research field of haematopoietic ageing will benefit from the development and application of single-cell metabolomics.

## Functional single-cell methods

Apart from purely sequencing-based methods, single-cell functional assays such as single-cell *in vitro* assays (Chapman et al., 2020; Lee-Six et al., 2018) or single-cell transplantations (Carrelha et al., 2018; Yamamoto et al., 2018) are also improving our understanding of haematopoietic ageing. In the landmark study by Lee-Six et al. (2018), single HSCs and HPCs from BM aspirate obtained from an adult man were expanded *in vitro* in single-cell liquid cultures. Subsequently, whole-genome sequencing of DNA isolated from single cell-derived colonies was performed to identify somatic mutations. In this setting, somatic mutations served as clonal markers to quantify the number, activity and longevity of human HSCs during normal haematopoiesis (Lee-Six et al., 2018). The same approach has been applied to study blood cells isolated from human foetuses, which shed light on the dynamics of foetal haematopoiesis (Chapman et al., 2020). Age-related functional changes in HSCs can also be successfully evaluated via single-cell transplantation assays (Yamamoto et al., 2018). Yamamoto et al. (2018) transplanted single HSCs isolated from young and old mice into lethally irradiated recipients and discovered a population of ‘latent HSCs’ exclusively in the aged BM. Latent HSCs showed a restricted myeloid potential in primary recipients but regained lymphoid potential when re-transplanted into secondary recipients. A better understanding of the development of latent HSCs could help in designing strategies to rebalance haematopoiesis in the elderly (Yamamoto et al., 2018).

## HSC transplantation at single-cell resolution

Advanced age is one of the risk factors for myeloid malignancies. HSC transplantation is a part of the standard treatment protocol for many haematological malignancies. Initially, this treatment strategy was restricted to younger patients, but its application in older adults has been increasing recently (Wildes et al., 2014). An interplay between the aged BM niche and blood reconstitution dynamics upon transplantation has been demonstrated (Ergen et al., 2012; Ho et al., 2019). Aged HSCs transplanted into young mice show improved capacity to generate lymphoid progeny, suggesting that the old BM microenvironment is involved in myeloid skewing (Ergen et al., 2012). Indeed, the absence of Notch delta-like ligand (*Dll4*) produced by BM vascular cells causes the premature upregulation of the myeloid transcriptional program in HSCs (Tikhonova et al., 2019). Single-cell-level analysis offers the opportunity to investigate context-dependent crosstalk between transplanted HSCs and BM cells. Tools like CellPhone DB (Efremova et al., 2020) enable the inference of cell-to-cell communication networks from scRNAseq data and can thus help to dissect the role of the microenvironment in myeloid skewing. Transplantation involves the subsampling of a genetically heterogeneous population of blood cells and then repopulation of the recently emptied BM niche. This subsampling of a genetically heterogeneous population creates an interesting genetic bottleneck that influences the dynamics of blood cell reconstitution. The increased occurrence of clonal haematopoiesis is one of the signs of haematopoietic ageing (Arends et al., 2018; Jaiswal et al., 2014). Several studies have investigated the impact of clonal haematopoiesis on the outcome of HSC transplantation therapy (Frick et al., 2019; Gibson et al., 2017; Mouhieddine et al., 2020; Ortmann et al., 2019). However, the implications of clonal haematopoiesis for stem cell transplantation are difficult to identify owing to the high degree of mutational heterogeneity present within the genetically distinct subclones (Park et al., 2019). Therefore, studying it at single-cell resolution can significantly improve our understanding of the process. The transplanted graft contains a heterogeneous population of cells, with only some of the cells harbouring clonal haematopoiesis-associated mutations. Advances in scRNAseq have revealed the transcriptional diversity among HSCs, thus providing a possible explanation behind their functional heterogeneity (Buenrostro et al., 2018; Giladi et al., 2018; Velten et al., 2017). However, the destructive nature of sequencing assays, which require cell lysis, prevents the simultaneous assessment of the transcriptome and functional output of the same cell, which is an important aspect from a transplantation point of view. This challenge was addressed by the application of lentiviral barcoding, which enables the simultaneous analysis of lineage outputs and transcriptomes from single HSCs during long-term bone marrow reconstitution in mice (Rodriguez-Fraticelli et al., 2020). Lentiviral barcoding has contributed to the identification of a mechanism for the maintenance of the self-renewing HSC state, which depends on transcription factor 15 (*Tcf15*) (Rodriguez-Fraticelli et al., 2020). This example highlights how the development of single-cell lineage-tracing methods, together with further application of single-cell techniques to study graft cells and blood cell reconstitution dynamics in patients, can bring us closer to better understanding the exact mechanisms active in the human body upon HSC transplantation. This knowledge could be translated into the optimisation of future transplantation strategies for elderly patients.

## Single-cell view on infections in elderly

Age-associated changes affecting the immune system are under extensive investigation in the clinic (Esme et al., 2019). The myeloid skewing of the blood compartment and immunosenescence occurring with advancing age can be associated with a decline in the adaptive immune response (Choudry and Frontini, 2016). With advancing age, infections become more frequent, are generally more severe and can present different symptoms than those in younger adults (Htwe et al., 2007). Infections are one of the leading causes of morbidity and mortality in people with advanced age (Esme et al., 2019). Seniors have an elevated risk of respiratory infections, such as severe influenza illness, which often requires hospitalisation (Nguyen et al., 2017). Advanced age is also considered a risk factor for severe outcome of *Clostridium difficile* infection (Shin et al., 2016). Finally, skin and soft tissue infections, e.g. with *Staphylococcus aureus*, in the elderly are frequently encountered both in hospitals and in the community (Laube and Farrell, 2002). Single-cell omics techniques allow the dissection of important cell-to-cell variation in the immune response that contributes to the infection outcome (Strzelecka et al., 2018). Accordingly, single-cell-level analysis of T cell receptors (TCRs) in cytotoxic T cells revealed that ageing was associated with reduced numbers of influenza-specific cytotoxic T cells (Nguyen et al., 2017). Recently, several groups applied a single-cell approach to understand the dynamics of SARS-CoV-2 (Schulte-Schrepping et al., 2020; Wen et al., 2020; Wilk et al., 2020; Zhang et al., 2020; Zheng et al., 2020). An interesting study deciphered the landscape of human circulating immune cells in young and old individuals and in COVID-19 patients (Zheng et al., 2020), applying scRNAseq, scATACseq and CyTOF to capture age- and COVID-19-associated changes at the transcriptomic, chromatin and protein level. The results revealed that SARS-CoV-2 infection amplifies the age-induced upregulation of inflammatory genes in immune cells, providing a possible explanation for the increased number of severe and fatal COVID-19 cases in elderly patients (Zheng et al., 2020). Single-cell approaches offer an in-depth understanding of the molecular changes underlying age-related immune dysfunction and can thus provide crucial insight into the development of elderly-targeted infection care.

## A glimpse into the future: concluding remarks

One of the biggest advantages of single-cell techniques is the broad readout of cellular composition from a relatively small amount of tissue. Genomic, transcriptomic and epigenomic technologies combined with proteome and metabolome analysis will generate multidimensional data with unprecedented insight into the ageing process. The ability to simultaneously detect changes at various molecular levels (epigenomic, transcriptomic, proteomic and metabolomic) is providing even more data to understand haematopoietic ageing. Each of these omics data can reveal useful markers of the process and provide insights into perturbed biological pathways. However, when analysed individually, omics data are just a snapshot of the processes happening within the cell and thus not sufficient to reveal the causal relationship between molecular signatures and the manifestation of a pathology. Integration of the different omics data types has the potential to uncover the causative changes underlying the age-associated phenotypes of blood cells.

### Single-cell techniques advancing haematopoietic ageing studies

There are several areas in which different single-cell techniques can make a unique contribution to haematopoietic ageing (Fig. 2). Currently, single-cell transcriptomics is the most widely used method (Ranzoni et al., 2019), followed closely by single-cell epigenomics (Kelsey et al., 2017). ScRNAseq has the power to detect ageing-related genes with limited expression level or expression restricted to a specific cell type. When combined with somatic mutation tracking (i.e. in clonal haematopoiesis studies), it can highlight changes that are directly linked to the mutation status of the cell (Nam et al., 2019). Analysis of population dynamics with scRNAseq precision can also uncover unique features such as the exhaustion of stem cells or cell population shifts (Fischer et al., 2019). Similarly, single-cell epigenetics approaches can reveal cell-type-specific epigenetic alterations emerging during ageing and its influence on gene expression patterns (Cheung et al., 2018). In a haematological context, the application of the multiomics approaches is already contributing to the better understanding of the human HSC compartment and the changes affecting immune cells (Granja et al., 2019). Further single-cell studies on the ageing blood system will help to answer the question whether altered signatures are indeed drivers or consequences of ageing. The global initiative of the Human Cell Atlas (HCA) (www.humancellatlas.org) is gathering multimodal single-cell data from various human tissues at an unprecedented scale. Comparison of the collected data between individuals of different ages will help to develop a geriatric atlas of the human body similar to Tabula Muris Sensis (The Tabula Muris Consortium, 2020). This will further advance our understanding of haematopoietic ageing.

### Limitations of single-cell techniques

Some limitations need to be taken into consideration when applying single-cell techniques in causative analyses to understand haematopoietic ageing. The majority of currently available scRNAseq platforms use poly(dT) primers (Box 1) to capture the poly(A) fraction of a cell's total RNA. This approach excludes non-coding RNA, which was shown to regulate the ageing processes (Sousa-Franco et al., 2019). Another drawback includes the identification of apparent changes in gene expression that are in fact caused by sample processing (van den Brink et al., 2017). To address this problem, new experimental protocols are being developed (Lambrechts et al., 2018), aiming to minimise gene expression alterations that are a consequence of experimental processing and that affect the downstream analysis of the sequencing data. There is also a specific problem related to the granulocytic population in droplet-based scRNAseq approaches. Granulocytes are characterised by relatively low RNA content and relatively high levels of RNases (Zilionis et al., 2019), which could degrade RNA during sample processing and thus interfere with the reliability of the resulting data. Therefore, supplementing buffers with RNase inhibitor might be beneficial when handling granulocyte samples. Additionally, during the data analysis step, a low filtering threshold for transcript count is recommended because granulocytes (especially neutrophils) can be inadvertently excluded when using the data filters commonly used in scRNAseq studies (Zilionis et al., 2019). Finally, analysing single-cell data from different platforms is a challenging task. Therefore, there is a constant need for the development of new computational methods capable of integrative data analysis. Currently available methods for single-cell multiomics data analysis have recently been reviewed in detail (Efremova and Teichmann, 2020). The development of single-cell multimodal omics tools will bring us closer to successfully dissecting the operational principles of biological systems.

### Bringing single-cell techniques to the clinical field

The future applicability of single-cell techniques in the clinical field will largely depend on the successful development of robust sample preprocessing techniques. Single-cell analysis in large clinical cohorts still remains expensive and faces technical difficulties. Several groups are putting an effort into benchmarking available protocols and optimizing the workflows for clinical settings to ensure the generation of valid high-quality results (Ding et al., 2020; Hanamsagar et al., 2020; Mereu et al., 2020). The development of compact single-cell work stations suitable for clinical laboratories will facilitate the application of single-cell approaches to clinical settings. Further advances in single-cell techniques will enable their transition from the bench to the bedside.

In conclusion, single-cell approaches keep refining our perception of haematopoietic ageing. In the future, integrative analyses of single-cell multiomics data will contribute to the creation of a detailed atlas highlighting age-associated alterations. This knowledge will have a direct potential to be translated into new geroprotective interventions benefiting the elderly population.
